# Search for the Profile of the Victim of Adolescent Dating Violence: An Intersection of Cognitive, Emotional, and Behavioral Variables

**DOI:** 10.3390/ijerph17218004

**Published:** 2020-10-30

**Authors:** Isabel Cuadrado-Gordillo, Inmaculada Fernández-Antelo, Guadalupe Martín-Mora Parra

**Affiliations:** Department of Psychology and Anthropology, University of Extremadura, 06071 Badajoz, Spain; iferant@unex.es (I.F.-A.); guadammp@gmail.com (G.M.-M.P.)

**Keywords:** dating violence, victim profile, moral disengagement, sexist attitudes, emotional dispositions, behavioral disorders

## Abstract

The knowledge of the promoting variables of dating violence has been a topic much studied in the last decade. However, the definition of the profile of this type of victim still presents numerous unknowns that hinder the effectiveness of prevention programs against violence. This study analyzes the interaction of cognitive, emotional and behavioral variables that converge in the victim profile. The sample comprised 2577 adolescents (55.2% girls) of 14 to 18 years in age (M = 15.9, SD = 1.2). The instruments used were the dating violence questionnaire (CUVINO), the scale of detection of sexism in adolescents (DSA), Mechanisms of Moral Disengagement Scale and Child and Adolescent Disposition Scale (CADS). To study the relationship between the different variables considered in this article, a SEM analysis was used. The results show that victims of gender violence and emotional abuse have high scores in benevolent sexism, moral disengagement and emotionally negative behavioral patterns. Likewise, the existence of an interdependent relationship between these three sets of variables was found.

## 1. Introduction

In recent years, ever more adolescents have found themselves involved in violent dating relationships [1,2]. These aggressive relationships are characterized by harassing behavior and physical, psychological, sexual and emotional aggression directed at the partner. The severity of this phenomenon worldwide has led it to come to be considered a public health problem [1,2,3] that is different from abusive adult relationships. Nonetheless, it has been found that the presence of violence in adolescent dating is often predictive of the likelihood of abusive adult relationships, thus aggravating its implications.

The development of violent behavior in dating relationships during adolescence has been linked over time to different variables such as the sexism and gender roles present in society. Glick and Fiske [4] developed the theory of ambivalent sexism, pointing to the coexistence of two forms of sexism in society: hostile sexism and benevolent sexism. Hostile sexism embodies male domination over females, while benevolent sexism is related to protective and affective attitudes directed towards the female, which leads to the devaluation of women. With regard to aggressiveness in dating relationships, benevolent sexism creates a greater acceptance of violence when it coincides with gender stereotypes [5]. Likewise, benevolent sexism makes many women favor the idea that they should be protected by their partners [6]. With this, some authors [7] found that victimization is frequently related to various types of sexism, while others [8] noted that high levels of benevolent sexism are associated with greater victimization in women, finding higher levels of verbal-emotional and sexual violence within the couple.

Likewise, it has been found that sexism influences aggression through its association with other variables. Thus, some authors [9] note the existence of correlations between such negative emotional variables as anger and sexism, which ultimately foster attitudes that are more favorable to behaviors such as rape [10] or other types of abuse [8].

However, beyond the sexism present in society, aspects such as adolescents’ attitude, behavior and conduct may also be linked to this violent phenomenon. Taking into account the appearance of aggressive behaviors, some authors have focused on exploring the variables associated with the development of conduct disorders during childhood and adolescence [11,12,13,14]. But there has been little research exploring how the development of these behavioral disorders might influence violence in adolescent dating and, more specifically, the role of the victims. This linkage could be especially relevant considering that some of the most notable characteristics of these behavioral disorders involve not only violent behavior but also harassment, threats and intimidation directed at others, physical cruelty and sexual violence [15], all aspects linked to violence within the couple.

Behavioral disorders or disturbances have been linked to the presence or absence of different variables during childhood and adolescence. Some of the most relevant are the development of audacious conduct (intense behaviors, a taste for danger, novelty and risk) and negative emotions (non-empathic responses, low feelings of guilt, little respect for rules, little concern for school work, etc.), as well as the absence of prosocial conduct (empathy, helpful responses, and concern for others).

Some of these traits increase the risk of such problems as violence, substance abuse, anxiety, depression, etc. [16,17]. Thus, some authors [12,18] note that adolescents who present high negative emotions, low prosociality scores, and the presence of audacious behavior, in combination with the influence of environmental and contextual factors, would have a greater predisposition to developing behavioral disorders. In contrast, those adolescents who show greater involvement in prosocial behavior are less likely to develop such behavioral disorders as aggressiveness or violence [19,20,21,22,23,24,25].

The positive influence that prosociality has on adolescents may, however, decrease due to the influence of cognitive variables that are also involved in the perpetration of violent behavior. In this regard, the use of moral disengagement mechanisms is a factor to be taken into account. Individuals who behave violently tend to use these mechanisms in order to cognitively reconstruct their behavior so that it seems less harmful or to justify their actions [26]. In this sense, there would be a tendency for these individuals to present a greater number of socially unacceptable behaviors, with a consequent reduction of prosocial conduct and concern for others [27,28]. Moral disengagement then becomes a variable that inhibits prosocial behavior [29,30]. In contrast, positive emotions and prosocial moral reasoning stand out for having a positive influence on the development of prosociality [31,32,33].

Finally, negative emotions have also been linked to violence in couple relationships through different channels. Researchers [34] note that individuals with high negative emotions and neuroticism are more likely to perpetrate aggressions within the couple than those with low levels of the said emotions. Other authors [35] confirm the strong relationship between negative emotions and the perpetration of physical violence, at the same time as revealing the protective value of positive emotions.

However, negative emotions do not usually occur alone. In fact, they tend to interact with other variables, with a resulting increase in aggressiveness and violence. Thus, models of social learning, as well as the cognitive-behavioral treatments used for interventions on negative emotions [36], suggest that these factors, together with other cognitive traits, lead people who are at risk of aggression to code and selectively interpret information, thereby increasing hostility, negative emotions, and consequently their aggressive responses [37]. Likewise, the influence of contextual factors such as stress could initiate a chain of negative elements that intensify the emotional experience, causing the levels of anger and negative emotions to increase until they become ill-adapted processes which raise the likelihood of responding in an aggressive or violent way when faced with having to resolve problems [38].

In summary, there is evidence that, in adolescence, the presence of audacious and risky behavior and a high negative emotionality are related to the development of violence. Likewise, prosocial behaviors seem to be protective factors that keep adolescents away from problematic behavior. However, these variables alone are not enough to explain the development of violent behavior, with it being common to find them interacting with other contextual, environmental and cognitive variables. With this, given the lack of research analyzing the possible implication that these traits may have for the phenomenon of adolescent dating violence, and, more specifically, for the victimization that is part of this phenomenon, the present research may contribute by analyzing the relationships that all these variables have with this type of violence. In this context, the focus of the present study was on analyzing how the presence or absence in adolescents of such risk variables as the profile of conduct disorder (prosocial behavior, audacious behavior, or negative emotionality) might interact with sexism or moral disengagement, influencing and moderating the victimization in adolescent dating relationships.

## 2. Materials and Methods

### 2.1. Participants

A total of 2577 adolescents (55.2% girls) between the ages of 14 and 18 (M = 15.9, SD = 1.2) participated in this cross-sectional study. The selection of this age range is for the moment in which the first relationships of couples begin and, therefore, the moment of early detection of situations of dating violence. These participants were selected following a stratified multistage, approximately proportional, sampling procedure with conglomerates and random selection of groups in public secondary schools in which compulsory secondary education (ESO) is taught. The strata considered were the provinces and geographical areas of Extremadura (Spain), selecting towns in the north, south, east, and west of the region, and taking their different socio-cultural contexts into account. Both rural and urban areas were included in the study. In urban areas, schools located in neighborhoods with high purchasing power where the students’ families have a medium-high salary were selected as part of the sample. But schools located in poorer neighborhoods where the families of the students have less purchasing power were also included. In rural areas, both the academic and economic level of the parents is usually significantly lower than in many urban areas. The income of these families is usually classified within the lower-middle level. The variety of socioeconomic status of the region was included with this kind of selection. Furthermore, in each of the defined strata, the election of towns and cities was proportional covering as much representativeness as possible. The conglomerates used were the secondary schools. In each school, one of the four courses (3rd and 4th secondary school, 1st and 2nd baccalaureate) was selected at random. The adolescent population of the region is approximately 24,000 people. Assuming a 95% confidence interval and a 2% margin of error, the sample size should be 2183 adolescents. In the present studio, in prevention of a high dropout rate or incomplete questionnaires, we established a desired sample of 2800 adolescents. The elimination of incomplete or invalid data, as well as the unwillingness to participate of some adolescents (dropout = 1.8 %, N = 50) reduced the sample to 2577 participants.

### 2.2. Instruments

Data collection was carried out through three questionnaires:

Dating Violence Questionnaire (Cuestionario de Violencia de Novios, CUVINO [39]. Through its first 42 items, this questionnaire makes it possible to identify adolescents who have been victims in their relationships according to a classification of eight forms of aggression: detachment, humiliation, sexual, coercion, physical, gender, emotional punishment and instrumental punishment (i.e., You feel forced to perform certain sexual acts). This identification also allows knowing the level of victimization frequency using a Likert scale of five values that go from ‘never’ (1) to ‘almost always’ (5). On the other hand, this instrument allows us to analyze the degree of acceptance or tolerance that adolescents show towards aggressions suffered or that they may suffer. Once again, these abuses are classified into eight modalities, coinciding with those noted above. A Likert scale of five values was used to measure the level of acceptance, ranging from the category ‘none’ (1) to ‘a lot’ (5). The reliability indices (Cronbach’s alpha) that this instrument registers with our study sample vary between 0.66 and 0.83 depending on the different violence subscales analyzed.

Mechanisms of Moral Disengagement Scale [40]. Through its 32 items, the score that adolescents achieve in each of the eight mechanisms of moral disengagement included in this instrument was analyzed: moral justification, euphemistic language, advantageous comparison, displacement of responsibility, diffusion of responsibility, distortion of consequences, attribution of blame and dehumanization. This instrument uses a five-value Likert scale with (1) ‘totally disagree’ and (5) ‘totally agree’ (i.e., It is alright to fight to protect your friends). The levels of reliability (Cronbach’s alpha) achieved in this sample range from 0.72 and 0.81 depending on the disengagement mechanism measured. On the other hand, these mechanisms can be grouped into four categories that facilitate their analysis. The level of reliability achieved in each of them is added in parentheses: behavioral locus (0.75), outcome locus (0.78), agency locus (0.79) and recipients locus (0.81).

Child and Adolescent Disposition Scale (CADS) [41]. The CADS had a response scale from not at all (1) to very much (4) and three subscales: negative emotionality, daring, prosociality (i.e., Wants everyone to follow the rules, including self). Negative emotionality is defined by items assessing frequent and intense negative emotional responses to frustrations, loss and threats. The prosociality scale quantifies caring about the welfare of others, spontaneous helping, attempting to please them, and experiencing guilt over misbehaviors. Children rated high on the *daring* scale find intense and risky situations to be attractive and rewarding. Daring is closely related to the constructs of sensation seeking. Alpha reliability for prosociality, negative emotionality and daring was 0.88, 0.81 and 0.83, respectively, in this sample.

Scale of Detection of Sexism in Adolescents [42]. This scale assesses the sexist attitudes that adolescents have towards traditional gender traits and roles, considering two sub-scales: hostile sexism and benevolent sexism. The hostile dimension refers to the traits and roles that place women in an inferior position. The benevolent dimension highlights the qualities of women related to raising and caring for the family. The scale used is a 6-point Likert type, with 1 being ‘totally disagree’ and 6 ‘totally agree’ (i.e., Women are, by nature, more patient and tolerant than men). The reliability analysis (Cronbach’s alpha) gave a value of 0.89 for the instrument overall, 0.91 for the hostile dimension, and 0.85 for the benign dimension.

### 2.3. Procedure

This study is part of a subsidized research project that previously required the approval of the Bioethics and Biosafety Committee of University of Extremadura (Spain) (Ref. 18/2017). Access to the schools required the authorization of the Management Teams with whom the researchers interviewed. However, this was not enough to initiate the data collection process. As it is a sample of adolescents (minors), in order to pass the questionnaires, authorization from the parents of the students was necessary. The researchers were the ones who went to schools to pass the paper questionnaires. Once inside the classroom, the researcher explained to the adolescents the objectives of the research, how data would be used, and the level of confidentiality assumed. Based on this information, teenagers could decide to participate or not participate in a free and informed manner. Those who did not want to participate remained in the classroom, but doing other activities. Finally, the researcher gave the questionnaires to each participant who had to fill them out individually. There was no compensation for participating, neither for adolescents nor for schools.

### 2.4. Analysis

The analyses are divided into three phases. In the first phase, the victims are identified and grouped according to types of aggression suffered, following the result of the cluster analysis described in [43]. In the second phase, correlation analyses are carried out to determine the relationship between the different study variables. In the third phase, a structural model is constructed through the SEM analysis carried out with the LISREL program. In this model, the victimization modalities are latent variables identified by the types of victimization analyzed, while the other measurements are included as observed variables. To analyze the moderating effect, the residual centering procedure was used to create interaction terms [44].

## 3. Results 

The descriptive (Table 1) and correlation analysis (Table 2) of the data reveals the association between the variables analyzed and the epidemiological values of the adolescents who participated.

Selected from the total sample are the subjects who declare themselves to be victims in their relationships (Table 3). The results show that, as the frequency of aggression increases, the number of victims decreases. But it is also found that those who self-identify as victims are not victims of a single type of abuse, but rather suffer poly-victimization.

For the construction of the structural model, hypothetical associations were made between sexism (benevolent and hostile), moral disengagement, behavioral profiles (prosociality, audaciousness and negative emotionality), and the three forms of victimization (emotional, gender and physical). To begin with, the model was tested only with the main effects, but the fitting values obtained were not satisfactory because some of the associations were not statistically significant: χ^2^(21) = 47.03, *p* < 0.001, χ^2^/*df* = 2.57, CFI = 0.94, GFI = 0.90, AGFI = 0.87, RMSEA = 0.08. Specifically, the profiles of prosociality and audaciousness, as well as hostile sexism, were not related to any of the victimization modalities, so these profiles and this type of sexism were eliminated from the model. However, the fit of the simplified model still did not present significant values: χ^2^(18) = 43.19, *p* < 0.001, χ^2^/*df* = 2.84, CFI = 0.96, GFI = 0.97, AGFI = 0.89, RMSEA = 0.07. On this occasion, it was detected that the relationships between negative emotionality and physical victimization, and between benevolent sexism and physical victimization, did not register statistically significant values, so these relationships were also eliminated from the model.

**Figure 1:**
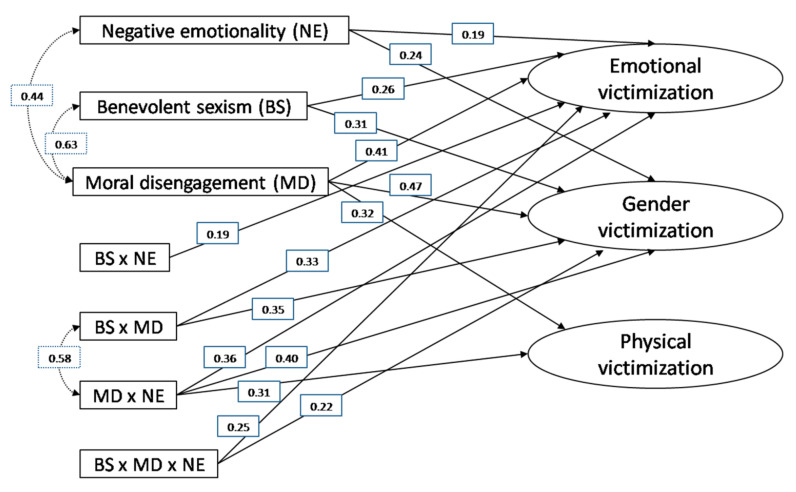
Final Structural Model.

In order to obtain a fuller model and to test the effects of moderation, various interaction terms were added. Some were two-way interactions: benevolent sexism and negative emotionality (BS×NE); moral disengagement and negative emotionality (MD × NE); benevolent sexism and moral disengagement (BS × MD). Another was three-way: benevolent sexism, moral disengagement and negative emotionality (BS × MD × NE). However, this model did not fit the data well: χ^2^(54) = 123.14, *p* < 0.001, χ^2^/*df* = 9.80, CFI = 0.79, GFI = 0.81, AGFI = 0.67, RMSEA = 0.18. Therefore, the statistically non-significant relationships were eliminated: specifically, the BS×NE interaction with gender victimization and physical victimization, the BS×MD interaction with physical victimization, and the BS×MD×NE interaction with physical victimization. Lastly, following the suggested modification indices, correlations between the exogenous variables were added (Figure 1).

This final model presented a satisfactory level of fit: χ^2^(25) = 53.42, *p* < 0.001, χ^2^/*df* = 1.60, CFI = 0.98, GFI = 0.97, AGFI = 0.94, RMSEA = 0.04. The results also showed that this model explains 62% of the variance of emotional victimization, 50% of the variance of gender victimization, and 34% of the variance of physical victimization.

With regard to the significant interactions, the follow-up analyses revealed that the positive relationship between EN and emotional victimization is stronger at high levels (+1 SD) of BS (β = 0.39, *p* < 0.001) than at low levels (−1 SD) of BS (β = 0.27, *p* < 0.1). It was also found that the positive relationships between BS and emotional and gender victimization were stronger at high levels (+1 SD) of MD (β = 0.43, *p* < 0.001 for emotional victimization and β = 0.41, *p* < 0.001 for gender victimization) than at low levels (−1 SD) of MD (β = 0.28, *p* < 0.01 and β = 0.26, *p* < 0.05 for emotional and gender victimization, respectively). Other results revealed that the positive relationship between EN and emotional victimization through BS is higher at high levels (+1 SD) of MD (β = 0.28, *p* < 0.001).

## 4. Discussion

The results found in the present study reveal the complex network of relationships that affects the victimization process in adolescent dating. In particular, varied and heterogeneous emotional, behavioral and cognitive factors act in combination to influence two of the main types of victimization: emotional victimization and gender victimization. Likewise, it was found that the impact of this combination of variables on victimization varies depending on the degree to which the adolescent victims present those characteristics, with high levels of benevolent sexism, moral disengagement, and negative emotionality being especially relevant in this regard.

With respect to the negative emotionality profile, it was found that the impact that this variable has on emotional victimization is greater when the victim presents a high level of benevolent sexism. These findings point in the same direction as previous studies that had already indicated how high negative emotionality, when interacting with other variables, might lead to an increase in an individual’s violent behavior [18,36,38,44,45,46,47]. However, it is important to note some differences from previous research studies. Thus, while authors such as [35] note the strong relationship between negative emotionality and physical aggression, the present results reveal a link between emotional victimization and negative emotions but do not find any relationship with respect to physical victimization.

A possible explanation for this finding could lie in the disparity between the violence occurring in adult relationships and that in relationships between adolescents. In this regard, it is important to note that the differential evolutive and generational level of the individuals involved in the two types of romantic relationship (adults and adolescents) complicates their comparison [48]. Thus, aspects such as that the couple not living together in the case of adolescents, and the greater use of information and communication technologies by this group [49], could indicate that the type of victimization in adolescents is more emotional than physical. This fact has also been verified in various studies which have noted that the most prevalent violence in adolescence is psychological [50,51]. Also, as noted in [52], several other factors such as emotional immaturity, unrealistic expectations of love, cognitive distortions, or conservative attitudes and perspectives about gender roles and sexism might be important in the differences found between adult and adolescent relationships and in the phenomenon of dating violence.

With regard to benevolent sexism, the importance of this factor in adolescent victims is evident. This highlights a fact that contrasts with some of the studies that have analyzed the role of benevolent sexism in violence within couples, the results of which have also been disparate. Thus, while some authors report inconclusive results [53,54,55], others have even been led to note that this factor could be a protective variable in dating violence directed towards women [56]. It is possible, however, that in the specific case of victimized adolescents who tend to maintain beliefs regarding the protection and care that women need, and who therefore present high levels of benevolent sexism, there is a certain degree of disappointment related to the non-fulfillment by their partners of the expectations they have created around the relationship.

During adolescence, beliefs in romantic myths of ideal love and in the existence of one’s other special half establish expectations that are very difficult to fulfill. This is especially so in girls, whose differential socialization still gives great importance to love, which even today can sometimes become the center of their life goals [57]. The possible disappointment that can come from the breach of these expectations may cause an increase in the levels of negative emotionality, and this in turn can intensify the selective coding of information which increases the likelihood of resolving problems through conflict [37], and thereby ultimately promoting emotional victimization. Something similar has been suggested by such authors such as [58] who indicate that adolescents identifying as victims tend to suffer from higher levels of negative emotions such as anger and sadness. Likewise, some authors [59] showed how the absence of good emotional regulation can cause an increase in adolescents’ vulnerability to victimization.

The absence of a connection between hostile sexism and the different types of victimization in adolescent dating relationships could again be explained by reference to the differences between these relationships and those of adults. Hostile sexism, which focuses on the importance of the dominance of men over women, the maintenance of traditional roles, and the treatment of women as sexual objects, seems to correspond to a lesser extent with today’s adolescents. Indeed, according to authors such as [60], this group of people tend to feel less identified with traditional male and female roles than do adults. This would also explain the prevalence of a benevolent sexism that, in today’s society, is magnified and spread through products of popular culture such as films, narratives, stories, songs, etc. [61]. Since women are exposed to these products to a greater extent, it would also hinder them from being able to recognize and fight against the said devaluation [62]. In this sense, many adolescent girls feel empowerment still to be a false construct [63] that gives them just an illusory feeling of freedom and equality, and this feeling allows them to continue to follow social conventions.

On the other hand, possible cultural differences should be considered. Related with this, it has been noted a higher social acceptance of violence against women in particular societies when comparing with others [64]. In this sense, dating violence could also be related with society’s perception of tolerance and acceptability [65,66,67,68]. Sexism seems to be similar and, as noted in [69], gender stereotypes are built around the cultural context. In that respect, it has been noted that Latin American children tend to be more traditional than White European American children, which in turn are more traditional than African American [70,71,72]. Consequently, minimizing sexism, and specifically benevolent sexism, seems to be an essential aspect which should be included both in prevention and intervention programs. In this sense, the concept of “Coeducation” considered as a process of intervention for boys and girls focused on the promotion of personal development and common social construction avoiding any opposition between the two different sexes [73] could be the key to achieving real equality for the future society. Schools and legislation should join to create a specific approach that addresses an integral, solid and real sexual [74] and affective education since the beginning of children’s development. On the other hand, addressing the false empowerment of adolescent girls should be a priority. Thus, recent works [75] have noted how education in preventing sexist beliefs and attitudes, in addition with good teaching practice, could take advantage of the use of technology and the new languages involved. In this sense, the success of apps such as “Liad@s” [76], which can be used both in recreational and learning environments has been pointed out, promoting reflection and learning regarding sexism and romantic love [75].

Another important finding of this study points to the absence of any relationship of prosocial behavior with the rest of the variables analyzed, i.e., negative emotionality, moral disengagement, benevolent sexism and physical, emotional, and gender victimization. In this regard, it is notable that, although it is true that prosocial behaviors have been established as protective factors that contribute to good emotional self-regulation [77], other authors [78] note how the link between emotion and cognition, and specifically the relationship regarding negative feelings centered on oneself, can lead to the perpetration of harmful behavior. Likewise, it has been found that the way in which adolescents handle such aspects as anger, excitability, emotionality and jealousy are predictors of violence in their dating [79,80]. The possible positive influence that prosocial behavior might have would thus be counteracted by the presence of negative feelings in the victims.

Finally, and linked to all of the above, moral disengagement seems to be an essential variable within the network of relationships found around the different types of victimization. Thus, the results show that adolescents with a high degree of use of these mechanisms show a stronger relationship between benevolent sexism and emotional or gender victimization. In general, many researchers have linked the use of these mechanisms to the perpetration of violent behavior [40,81,82,83]. Likewise, other studies [84] add how moral disengagement allows the aggressions suffered to be reconstructed so that they can be perceived as less harmful. The victim’s point of view, however, has been less explored. It has not been until recent research [85] that the way in which these mechanisms interact with other cognitive variables has been highlighted as one more strategy with which to minimize the importance of victimization. It is thus likely that the interaction found between moral disengagement, benevolent sexism, and emotional and gender victimization is one more interaction to take into account within the phenomenon of victimization.

From this perspective, it is possible that victims use these mechanisms not only to diminish the importance of the aggressions, but also as an element that helps hide and minimize the stereotypes linked to benevolent sexism, which in turn is related to greater emotional and gender victimization. Benevolent sexism is considered to be a type of sexism that presents a positive affective tone directed towards the stereotyped feminine ideal of women as good wives, mothers and sentimental partners [86]. It could be linked to victimization both in its emotional aspect, in which the victims are mistreated through such behaviors as humiliation or disparagement that provoke strong anxiety reactions [87], and in its gender aspect, in which violence is exerted due to the specific and subjective difference between the sexes and causes different types of harm to the victims [88]. As a result, following some authors [89] one could say that moral disengagement influences the victims not only through minimizing the importance of the aggressions suffered, but also by justifying the absence of actions on their part aimed at breaking the victimization situation. The result of all the relationships found would then give rise to a network that is difficult to break, which acts by feeding itself reciprocally and continually, greatly complicating the solution of the problem, and in many cases causing the victimization to continue over time.

## 5. Limitations

This study has some limitations. First, it is a cross-sectional study, so that there must be caution in making any generalization of the results or in determining any causal and predictive relationship. Second, the analysis did not take age into account as a variable. The evolutionary moment of the teen years (14–18) might have some influence on the results. Aspects such as moral disengagement, sexism or negative emotionality have different characteristics at the end of the adolescence than in the mid or early adolescence. Additionally, the evaluation of the interpretations of offenders could add a wider perspective to the results.

These limitations should serve to orient the direction of new research focused on gaining deeper knowledge of the phenomenon of dating violence and victimization. This way, longitudinal studies could contribute to covering the different evolutionary moments in adolescence.

## 6. Conclusions

Adolescent dating violence has been the subject of diverse studies in different places over time. The general trend of the research has been to focus on the aggressors, attempting to discover the reasons that led these individuals to perpetrate violent behavior and harassment directed at their romantic partners. The greatest contribution of the present study is, therefore, in offering an alternative perspective, one focused on the victims, and in considering a greater number of variables that interact with each other to give rise to adolescent victimization.

The victims of violence in adolescent dating relationships, far from being influenced by just one or two variables that explain the situation, are involved in a network of interactions that jointly influence their behavior, thus promoting the initiation and maintenance of this type of abusive relationship. It has been possible to verify how key factors, both emotional and cognitive, join together to create and moderate a complex web of interactions. Thus, it was revealed that the variables negative emotionality, benevolent sexism, and moral disengagement moderate emotional and gender victimization in adolescents. Specifically, it was observed that negative emotionality is linked to emotional victimization, with both variables in turn being influenced by high levels of benevolent sexism. A high degree of moral disengagement is also related to emotional and gender victimization, as well as to benevolent sexism. However, it is found that other factors traditionally linked to violence in partner relationships are ruled out in the case of today’s adolescents, for whom the influence of hostile sexism does not seem to be relevant.

These findings thus not only characterize more precisely the phenomenon of adolescent dating violence and of the victims involved in these relationships, but also open the door to the development of prevention, detection and intervention protocols that better match the reality of adolescents’ lives today.

## Figures and Tables

**Table 1:** Statistical descriptions according to gender.

Variables	Range	Full Sample	Boys	Girls	*t* Test Gender	d
1. Emotional victimization	1–5	1.97 (0.23)	1.95 (0.22)	2.01 (0.25)	1.19	0.14
2. Gender victimization	1–5	1.84 (0.32)	1.65 (0.24)	2.03 (0.42)	2.14 *	0.26
3. Physical victimization	1–5	1.54 (0.44)	1.42 (0.40)	1.63 (0.49)	1.98 *	0.24
4. Moral disengagement	1–5	2.86 (0.26)	2.76 (0.28)	2.94 (0.23)	1.24	0.16
5. Benevolent sexism	1–6	2.94 (0.49)	2.83 (0.53)	3.06 (0.46)	1.22	0.15
6. Hostile sexism	1–6	1.82 (0.41)	1.78 (0.38)	1.89 (0.44)	1.06	0.12
7. Prosociality	1–4	2.73 (0.42)	2.68 (0.39)	2.77 (0.44)	0.98	0.10
8. Audaciousness	1–4	2.33 (0.67)	2.51 (0.78)	2.15 (0.54)	2.12 *	0.25
9. Negative emotionality	1–4	2.60 (0.49)	2.54 (0.48)	2.63 (0.51)	0.70	0.08

* *p* < 0.05.

**Table 2:** Correlations in all the sample.

Variables	1	2	3	4	5	6	7	8	9
1. Emotional victimization	-								
2. Gender victimization	0.12	-							
3. Physical victimization	0.09	0.14	-						
4. Moral disengagement	0.24 **	0.22 **	0.27 **	-					
5. Benevolent sexism	0.29 ***	0.19 **	0.27 **	0.32 ***	-				
6. Hostile sexism	0.07	−0.15 *	−0.09	0.39 ***	0.14 *				
7. Prosociality	0.02	−0.17 *	−0.19 *	−0.34 ***	−0.13	−0.18 **	-		
8. Audaciousness	0.11 *	0.06	0.14 *	0.21 **	0.05	0.16 **	0.16 **	-	
9. Negative emotionality	0.23 **	0.19 **	0.15 *	0.26 **	0.15 **	0.12 *	−0.08	0.16 **	-

* *p* < 0.05; ** *p* < 0.01; *** *p* < 0.001.

**Table 3:** Frequency and modalities of victimization.

Victimization	Frequently	Usually
Victims	395 (15.33%)	91 (1.59%)
Detachment	258	50
Humiliation	82	10
Sexual	73	20
Coercion	141	40
Physical	34	9
Gender-Based	90	19
Emotional Punishment	188	42
Instrumental	42	6
